# Early maladaptive schemas are associated with self-injury thoughts and behavior in adolescents

**DOI:** 10.1186/s12888-023-05127-7

**Published:** 2023-08-29

**Authors:** Pauliina Saarijärvi, Christina Salmivalli, Saija Helmi, Max Karukivi

**Affiliations:** 1 Department of Psychology and Speech-Language Pathology, University of Turku, Publicum building, 4th floor, 20014 Turku, Finland; 2 Department of Psychiatry, HUS Helsinki University Hospital and University of Helsinki, Adolescent Psychiatry, Finland; 3 Psychiatric Care Division, Satasairaala Hospital, Pori, Finland; 4 University of Turku, INVEST Research Flagship, Publicum building, 4th floor, 20014 Turku, Finland; 5 Faculty of Social Sciences (SOC), Tampere University, Welfare Sciences, Psychology, 33014 Finland; 6 Department of Adolescent Psychiatry, University of Turku and Turku University Hospital, Kunnallissairaalantie 20, Turku, 20700 Finland

**Keywords:** Adolescents, Early maladaptive schemas, Self-harm, Self-injury, Young Schema Questionnaire, Ottawa Self-Injury Inventory

## Abstract

**Background:**

Early maladaptive schemas (EMSs) and self-harm have been firmly linked in adults, but research on these associations in adolescents remains scarce. Additionally, the links between EMSs and functions of self-injury has not been previously studied in this age group. Thus, the aim of the present study was to investigate the associations of EMSs with self-harm thoughts and behavior, as well as with self-harm functions, among adolescents in specialized health care.

**Methods:**

The participants were recruited from first-visit 12-22-year-old adolescent patients entering specialized mental health care or pediatric care. For 118 participants, complete data were available for the Young Schema Questionnaire Short Form 2-Extended (YSQ) when entering care and the Ottawa Self-Injury Inventory Functions scale (OSI-F) one year later. YSQ was used to measure the participants’ EMSs and OSI-F their self-harm thoughts and behavior. The associations of EMSs and self-harm were investigated in three groups: no self-harm, self-harm thoughts only, and both self-harm thoughts and behavior. The associations of EMSs with self-injury behavior functions were assessed in four categories: Internal Emotional Regulation, External Emotional Regulation, Social Influence, and Sensation Seeking. Additionally, EMSs’ associations with addictive features of self-injury behavior were assessed. The magnitudes of effect sizes of differences between the self-harm groups were evaluated with Cliff’s Delta. The associations of EMSs with self-injury functions were analyzed with general linear modeling and with self-injury addictive features using logistic regression.

**Results:**

The differences between the self-harm groups were significant for the majority of the EMSs. The stronger the EMSs were, the more severe the manifestations of self-harm. The effect sizes ranged from small to large depending on the EMS. Considering self-injury functions, Internal Emotional Regulation was associated with Self-Sacrifice EMS (p = 0.021), and External Emotional Regulation both with Abandonment (p = 0.040) and Unrelenting Standards (p = 0.012) EMSs. Being addicted to self-injury was associated with Abandonment (p = 0.043) and Dependence (p = 0.025) EMSs.

**Conclusions:**

The present study shows that significant associations between EMSs and both self-harm thoughts and behavior exist also in adolescents. Stronger EMSs are linked to more severe self-harm. Knowledge of these associations may help to improve the understanding and treatment of adolescents suffering from self-harm.

## Background

This study aims to investigate the associations of early maladaptive schemas (EMSs) with both suicidal self-harm and non-suicidal self-injury in adolescence. Self-injuring and suicidal behavior, which does not lead to death comprises suicide attempts, preparations for suicide, and interrupted or self-discontinued attempts of suicide [1]. Conversely, ’Nonsuicidal self-injurious behavior’ (NSSI) refers to intentional and self-caused harm to one’s own body without an aim of dying [1]. It has been estimated that 9.7% of adolescents have attempted to commit suicide and 29.9% have had suicidal thoughts at some phase in their life [2]. In addition, the average lifetime prevalence rates for self-harm and self-injury are 16.1% and 18.0% [3].

Several theoretical frameworks and assessment methods in understanding self-harm and especially suicidality have been developed [4–9]. They have been utilized also among adolescents [6, 10, 11]. Considering self-harm without suicidal intent, research among young people has shown that fluctuations in affective states can predict having thoughts of self-harm, but not engaging in self-harming acts [12]. During urges, which lead to harming oneself, thoughts of hopelessness and criticism towards oneself are experienced as the most upsetting [13]. Those who engage in self-harm ideation and behavior have elevated judgmental attitude towards themselves, more excessive identification and more isolation [14].

Research on adolescents has demonstrated that the functions of self-harm behavior reflect both intrapersonal as well as interpersonal features [15, 16]. However, it seems that intrapersonal functions, also referred as ’automatic reinforcements’, are more common [17, 18] and more pertinent [19] than interpersonal functions, as in ‘social reinforcements’ [17]. Although automatic reinforcements are most often reported as inducing self-harm [20], there is also evidence that both social and automatic reinforcements have significance to self-harm [21]. Indeed, the association of emotion regulation with self-harm has been repeatedly shown to exist [22], also in adolescents [23–26].

One more comprehensive model in understanding the functions of self-injury includes four behavioral functions, as well as addiction characteristics of self-injury [27–29]. The model comprises functions of Social Influence (attaining or changing something in social environment), Sensation Seeking (attaining feelings of, e.g., excitement), Internal Emotion Regulation (regulating internalized emotional experiences, e.g., sadness) and External Emotion Regulation (coping with emotions which could be externally expressed otherwise, e.g., anger) [27–29]. In adolescents, elevated frequency of self-injuring has been connected to elevated scores on all the functions apart from External Emotion Regulation, and addiction characteristics to an increased frequency of the behavior [27]. In young people, elevated endorsement of External Emotion Regulation, Internal Emotion Regulation, Sensation Seeking, and addiction characteristics have been connected with an increased lifetime frequency of self-injurious behavior, more experiences of distress associated with urges to it and with recent self-injury [29]. Self-injuring behavior in adolescents with higher internalized anger may be associated with more addiction traits [30].

Early maladaptive schemas (EMSs) are pervasive and extensive models of functioning regarding relationships with other people and oneself [31]. EMSs are by nature detrimental, and although they develop in youth or childhood, they function in adulthood as well [31]. EMSs are formed as a result of harmful experiences and unmet emotional needs in childhood, and they activate later in life when experiences reminiscence of, or similar to, those experiences are faced [31]. However, individuals often do not recognize that their interpretations no longer correspond with the reality of the situations [31]. Thus, maladaptive means to cope with EMSs are learned, and EMSs and those means form a core for various psychological problems [31]. The 18 EMSs and the current four-domain schema model [32] are presented in Table 1.

**Table 1:** Early maladaptive schemas (EMSs) and the four schema domains

EMS	Schema domain
Emotional Deprivation	Disconnection and Rejection
Social Isolation	
Emotional Inhibition	
Defectiveness	
Mistrust/Abuse	
Negativity/Pessimism	
Dependence	Impaired Autonomy and Performance
Failure to Achieve	
Subjugation	
Abandonment	
Enmeshment	
Vulnerability	
Self-Sacrifice	Excessive Responsibility and Standards
Unrelenting Standards	
Punitiveness	
Entitlement	Impaired Limits
Approval-Seeking	
Insufficient Self-Control	

Research on EMSs in adolescence has been quite scarce, but there is some evidence that EMSs are related to self-harming behavior [33]. Elevations in Emotional Inhibition, as well as Social Isolation, in addition to lower Entitlement have been associated with self-harm [33]. Additionally, Emotional Deprivation, Insufficient Self-Control, Mistrust/Abuse and Social Isolation may differ between those with and without self-harm [34]. Perceived greater rejection from parents has been shown to connect with greater endorsement of EMSs and with interpersonal and intrapersonal self-harming behavior motivations [35]. Both Emotional Deprivation and Defectiveness may be relevant treatment targets in preventing suicide [36], as EMSs may mediate the link between socioenvironmental aspects and adolescents’ suicidality and mood [37].

As summarized in this section, previous studies support the existence and relevance of the associations of EMSs with self-harm and self-injury. Although some studies indicate that these associations exist in adolescence as well, research on these connections has so far been scarce. In particular, EMSs’ associations with the NSSI functions and addiction features have been scarcely studied. In addition to increasing the knowledge on these themes, it is important to gain a deeper understanding on self-injuring behaviors in adolescence, so that more sophisticated and comprehensive treatment methods in clinical practice could be developed. In the present study the term ’self-harm’ (SH) refers to both suicidal and non-suicidal self-harm, and ’non-suicidal self-injury’ (NSSI), or in short, ‘self-injury’, refers to self-injuring behaviors without suicidal intentions. We hypothesized that EMSs differ in their associations with SH/NSSI thoughts and behavior, as well as with NSSI functions and addiction features. The present study aims to address the following questions:

1) Do EMSs associate with self-harm in adolescents who (a) have not had self-harm thoughts or have not engaged in self-harm behavior, (b) have had self-harming thoughts but have not engaged in self-harming behavior, and (c) have had both self-harming thoughts and have engaged in self-harm behavior?

2) Do EMSs have associations with each function of adolescents’ non-suicidal self-injury behavior?

3) Do EMSs have associations with the addictive features of adolescents’ non-suicidal self-injury behavior?

## Methods

### Participants

The study was completed as a part of the research project ’Emotions and well-being in adolescents’, for which participants had been recruited from first-visit patients referred to specialized health care in Satakunta Hospital District, Finland. The initial study sample comprises two separate samples: one recruited from adolescent psychiatric outpatient clinics and one recruited from the pediatric outpatient clinic. In adolescent psychiatry, the target group for recruitment included all 13–22-year-olds referred to the outpatient clinics in the region. In the pediatric clinic, the recruitment target group comprised 12 − 16-year-old patients. In order to have a sample representing actual patient population as accurately as possible, these age ranges were based on the age range of adolescents the clinics serve. Additionally, there were no tight inclusion or exclusion criteria: all patients coming to their first visit to the clinics were offered the possibility of participating in the study. The recruitment process extended from November 2017 to January 2019 (T1), and the whole study project sample comprised 309 adolescents. All participants gave a written informed consent and for those aged under 15, their guardians also gave their consent. The study protocol was approved by the Ethics Committee of the Hospital District of Southwest Finland (ETMK 89/1801/2017).

**Fig. 1:**
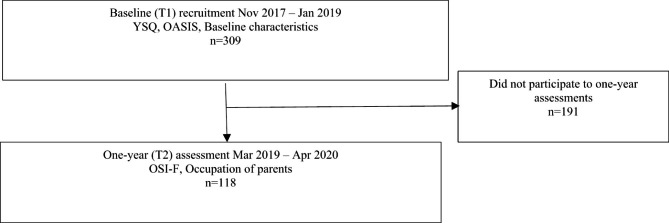
Recruitment of the participants in the whole research project and in the present study. YSQ = Young Schema Questionnaire-Short Form 2-Extended, OASIS = Overall Anxiety Severity and Impairment Scale, OSI-F = the Ottawa Self-Injury Inventory – Functions - v3.1

For the present study, the participants were contacted one year after entering the study, and the recruitment for the present study took place between March 2019 and April 2020 (T2). All participants who had participated in the project at T1 were offered the possibility to participate in the present study, resulting in a sample of 118 adolescents (from now on, ‘participants’). The recruitment process is presented in the flow chart (Fig. 1). Since the measurements were not repeated, the study setting is cross-sectional.

Of the 118 participants, 86 were female and 32 male. The age of the participants measured at T1 varied between 12 and 22 years (Median = 15.00, IQR = 4.00). The median time gap between T1 and T2 was 409 days (IQR = 79.00) and thus, the participants were on average 16 years old at T2. Attrition analyses based on the T1 measurements showed that those who participated in the present study did not differ from non-participants regarding their patient group (adolescent psychiatry vs. pediatric), age, living conditions, occupation, or anxiety score as measured using the Overall Anxiety Severity and Impairment Scale (OASIS) [38]. However, the rate of females participating in the present study was higher (χ^2^ [1] = 4,755, p = 0.029), and the rate of those who had no experience of using tobacco products was higher in the participating group (χ^2^ [1] = 3,871, p = 0.049). There were also differences between those who participated in this study and those who did not as regards their Young Schema Questionnaire – Short Form 2-Extended (YSQ) [39] scores on the following EMSs: Emotional Deprivation (Median = 2.00, IQR = 1.63 vs. Median = 1.60, IQR = 1.48, p = 0.028), Abandonment (Median = 3.00, IQR = 2.60 vs. Median = 2.40, IQR = 2.40, p = 0.025), Self-Sacrifice (Median = 3.60, IQR = 1.60 vs. Median = 3.30, IQR = 1.80, p = 0.037), Emotional Inhibition (Median 2.40, IQR = 2.00 vs. Median = 1.80, IQR = 1.40, p = 0.003), and Unrelenting Standards (Median 3.20, IQR = 2.00 vs. Median = 2.60, IQR = 2.00, p = 0.006). The participants had higher scores than the non-participants.

### Measures

EMSs were assessed using the YSQ scale [39]. The version of the YSQ implemented in this study contains 90 items and 18 schemas, and each of the EMSs comprises five items that are scored on a scale of 1 to 6 (1 = does not suit me at all … 6 = suits me perfectly) [39, 40]. To reach a score for each EMS, an average score is counted for the five items [39, 40]. The factor structure of the questionnaire has been studied to be comparable with the structure of 18 EMSs [40]. The YSQ has been implemented in studies (with different versions of the scale) with adolescents as well, and the results have shown that EMSs are also verifiable among individuals in their preadolescence-adolescence [41, 42]. For each of the EMSs, participants with at least 80% (4/5) completed items were included in the analyses. The missing values were estimated, using the mean value of the answered items. YSQ was filled at T1.

Self-harm and self-injury were assessed using the Ottawa Self-Injury Inventory – Functions – v3.1 (OSI-F) questionnaire [27, 43]. The questionnaire first assesses the features of both suicidal and non-suicidal self-injuring thoughts and behaviors, e.g., how often during the past month/6 months the individual has thought about or injured themselves (range 0–3 or 0–4, 0 = not at all, … 3 or 4 = daily), how often during the past year they have thought about suicide (range 0–4, 0 = not at all, … 4 = daily), and whether or not they have ever tried to kill themselves, or if they have ever needed treatment or hospitalization due to their self-injurious behaviors [27, 43]. The questionnaire also assesses the functions of NSSI behavior (25 questions, range 0–4, 0 = never a reason … 4 = always a reason) from the perspective of four functions: Social Influence (9 items), Sensation Seeking (4 items), Internal Emotion Regulation (8 items), and External Emotion Regulation (3 items) [27, 28, 43]. The score of each function is based on a mean score of the items in the function [27, 28, 43]. OSI-F also presents an addiction-related features component (7 items, range 0–4, 0 = never, … 4 = always), and if an individual scores ≥2 on three or more items, the threshold for an addictive component in self-harm is met [27, 28, 43]. Psychometric properties of the questionnaire have been studied in adolescent and young adult samples and OSI-F’s reliability and validity has attained support [27–29]. The OSI-F questionnaire was translated from English to Finnish by two of the authors (PS and MK), and the back-translation into English was performed by independent professional linguists. The Finnish translation was then approved by the developer of the instrument. OSI-F questionnaire was completed at T2.

In order to assess the association of EMSs with adolescents’ self-harming thoughts and behavior, the participants were assigned into three categories based on their answers in the OSI-F [27, 43] regarding the status and history of their self-harm (both suicidal and non-suicidal): (I) those who have not had self-harm thoughts or engagement in self-harm behavior (No self-harm, NSH), (II) those who have had self-harm thoughts but no engagement in self-harm behavior (Self-harm thoughts but no behavior, SHT), and (III) those who have had self-harm thoughts and engagement in self-harm behavior (Self-harm thoughts and behavior, SHTB). Additionally, in order to analyze the significance of EMSs on the addictive features of participants’ NSSI, the participants were divided into two categories based on whether the criteria for addiction were met in the Addiction Component of the OSI-F scale [27, 43] or not (Addicted/Not Addicted).

Participants’ anxiety was assessed using the OASIS questionnaire [38]. OASIS measures with five items (range 0–4, total score range 0–20) impairment related to anxiety and anxiety severity, and it has been found to be a valid and reliable measure [38]. In the present study, the OASIS score was used for controlling confounding effects of anxiety for our main study variables. The OASIS questionnaire was completed at T1.

Since both EMSs and SH/NSSI are related to sociodemographic factors, several variables gathered at T1 and T2 were included in the analyses in order to avoid overestimating the significance of the associations: participants’ age (T1), living conditions (with biological parents/with family of one biological parent/alone or other) (T1), smoking tobacco/using snuff (never tried either/irregular or regular use) (T1), occupation (lower secondary school/upper secondary school or higher education student/other) (T1), and occupation of their parents (both working/only one or neither working) (T2).

### Statistical methods

The normality of the distributions of the continuous variables was assessed both graphically and with the Shapiro-Wilk Test. Due to their non-parametric distributions, the variables are characterized using medians and interquartiles (IQR).

The correlation analyses for the association of the YSQ and OSI-F function scores, as well as the OASIS scores, were performed using Spearman’s correlation. Categorized variables were compared using the Chi-Square tests. For continuous variables, group differences were analyzed using the Mann-Whitney U Test for comparisons between two groups and the Kruskall-Wallis Test for three groups. The effect size for the score difference was estimated using Cliff’s Delta, also known as rank biserial correlation [44]. The following values for the magnitude of the effect size were used: ≥0.15 small, ≥0.33 medium, and ≥0.48 large. These correspond to Cohen’s d values of ≥0.20 for small, ≥0.50 for medium, and ≥0.80 for large effect size [45].

In order to analyze the associations of EMSs and non-suicidal self-harm functions, correlational analyses were completed on all 18 EMSs and all four OSI-Functions (Internal Emotional Regulation, Social Influence, External Emotional Regulation and Sensation Seeking) of adolescents’ NSSI. In addition, correlations of EMSs and OSI-Functions were also analyzed with age and the OASIS total score.

The significance of the YSQ scores at predicting the OSI-F function scores was assessed using general linear model (GLM). The fit of the models was evaluated based on the normality and variance of the residuals. Possible multicollinearity was assessed with the variance inflation factor and condition index, and no multicollinearity was observed. The residuals for the OSI-F Internal Emotion Regulation score showed a good fit and thus, the variable was used as parametric in the GLM analysis. For the OSI-F Social Influence, External Emotion Regulation, and Sensation Seeking, log-transformed values were used in order to meet the assumption of normality in the analyses. The GLM analyses included the variables that were significantly (p < 0.05) associated with the OSI-F function scores in the univariate analyses. The significance of the YSQ scores at predicting the Addictive Features function in the OSI-F scale was assessed with logistic regression. The logistic regression analyses included the variables that were significantly (p < 0.05) associated with the Addictive Features function in the univariate analyses.

The internal consistency for YSQ, OSI-F function scores and OASIS was calculated using Cronbach’s alpha. The Cronbach’s alpha scores were as follows: OASIS 0.906, OSI-F Internal Emotional Regulation 0.795, OSI-F Social Influence 0.589, OSI-F External Emotional Regulation 0.462, OSI-F Sensation Seeking 0.498 and all the scores for the EMSs ranged from good to excellent internal consistency: 0.707–0.968. In all analyses, p-values < 0.05 were considered statistically significant. Statistical analyses were carried out using the IBM SPSS software, Version 25.0.

## Results

### Demographic characteristics

Of the 118 participants, 79 were recruited from adolescent psychiatric outpatient clinics and 39 from the pediatric outpatient clinic. Most participants lived with their biological parents (n = 61), and most of the participants (n = 62) were pupils in lower secondary school. The majority of the participants had not tried tobacco products (n = 72), and the majority of the participants’ parents were both working (n = 81). Participants’ OASIS total scores varied between 0.00 and 17.00 (Median = 9.00, IQR = 9.00). Participants’ sociodemographic factors are summarized in Table 2.

**Table 2:** Descriptives and distributions of the participants’ sociodemographic factors by gender

		Females (n = 86)	Males (n = 32)	All (n = 118)		
Variable		n (%)	n (%)	n (%)	χ^2^	p
Patient group	Adolescent Psychiatry	59 (68.6)	20 (62.5)	79 (66.9)	0.393^a^	0.531^a^
	Pediatrics	27 (31.4)	12 (37.5)	39 (33.1)		
Dwelling	With biological parents	43 (50.6)	18 (58.1)	61 (52.6)	0.582^a^	0.748^a^
	With family of one biological parent	24 (28.2)	8 (25.8)	32 (27.6)		
	Alone or other	18 (21.2)	5 (16.1)	23 (19.8)		
Occupation	Lower secondary school pupil	44 (51.2)	18 (56.3)	62 (52.5)	6.778^a^	0.034^a^
	Upper secondary school or higher education student	26 (30.2)	3 (9.4)	29 (24.6)		
	Other	16 (18.6)	11 (34.4)	27 (22.9)		
Smoking or snuffing tobacco	Never tried either	50 (58.1)	22 (71.0)	72 (61.5)	1.584^a^	0.208^a^
	Irregular or regular use	36 (41.9)	9 (29.0)	45 (38.5)		
Occupation of parents	Both working	56 (67.5)	25 (78.1)	81 (70.4)	1.259^a^	0.262^a^
	Only one parent or neither working	27 (32.5)	7 (21.9)	34 (29.6)		

^a^Chi-Square test, comparison between the genders

### Associations and differences of EMSs to adolescents’ self-harm thoughts and behavior

When the scores on all 18 EMSs were compared between the self-harm groups, the SHT group had higher scores than the NSH group for EMSs of Emotional Deprivation, Abandonment, Mistrust/Abuse, Social Isolation, Defectiveness, Failure, Vulnerability, Subjugation, Self-Sacrifice, Emotional Inhibition, Unrelenting Standards, Insufficient Self-Control, Negativity/Pessimism and Punitiveness (Table 3). The SHTB group scored higher on all EMSs except Unrelenting Standards and Approval-Seeking when compared to the NSH group. The SHTB group also had higher scores than the SHT group in EMSs of Emotional Deprivation, Mistrust/Abuse, Social Isolation, Defectiveness, Failure, Dependence and Insufficient Self-Control.

**Table 3:** Comparisons of Young Schema Questionnaire scores between the self-harm behavior groups

	No self-harm thoughts or behavior (I)		Self-harm thoughts, but no self-harm behavior (II)		Both self-harm thoughts and behavior (III)		Group comparisons			
	Median	IQR	Median	IQR	Median	IQR	Comparison	U^a^	p^a^	Effect size^b^
Emotional deprivation	1.20	1.00	1.90	1.85	2.80	1.40	I vs. II	932.500	0.002	-0.42
							I vs. III	1506.000	< 0.001	-0.67
							II vs. III	899.500	0.039	-0.28
Abandonment	1.60	2.00	3.30	2.85	3.80	1.75	I vs. II	967.500	0.001	-0.47
							I vs. III	1432.500	< 0.001	-0.59
							II vs. III	796.500	0.330	-0.13
Mistrust/Abuse	1.20	0.90	2.20	1.80	2.80	1.80	I vs. II	921.500	0.003	-0.40
							I vs. III	1459.500	< 0.001	-0.65
							II vs. III	878.000	0.041	-0.26
Social Isolation	1.60	1.20	2.60	1.95	3.40	1.40	I vs. II	904.500	0.006	-0.38
							I vs. III	1498.000	< 0.001	-0.66
							II vs. III	954.500	0.008	-0.36
Defectiveness	1.00	0.70	2.00	2.20	3.40	2.00	I vs. II	967.000	< 0.001	-0.52
							I vs. III	1509.500	< 0.001	-0.71
							II vs. III	879.500	0.019	-0.32
Failure	1.50	1.40	2.40	2.40	4.00	2.55	I vs. II	828.500	0.015	-0.34
							I vs. III	1445.000	< 0.001	-0.64
							II vs. III	960.500	0.003	-0.41
Dependence	1.20	0.90	1.60	1.15	2.40	1.90	I vs. II	787.000	0.139	-0.20
							I vs. III	1237.500	0.001	-0.44
							II vs. III	895.500	0.014	-0.33
Vulnerability	1.40	0.80	2.17	1.40	2.50	1.70	I vs. II	1001.000	< 0.001	-0.53
							I vs. III	1326.000	< 0.001	-0.54
							II vs. III	743.000	0.438	-0.11
Enmeshment	1.00	0.70	1.40	0.80	1.60	1.00	I vs. II	773.500	0.102	-0.22
							I vs. III	1202.000	0.003	-0.43
							II vs. III	789.500	0.173	-0.25
Subjugation	1.00	0.80	2.20	1.80	2.60	1.80	I vs. II	944.500	< 0.001	-0.49
							I vs. III	1430.500	< 0.001	-0.62
							II vs. III	800.000	0.143	-0.20
Self-Sacrifice	2.97	1.95	3.60	1.80	4.20	1.35	I vs. II	856.500	0.006	-0.38
							I vs. III	1281.000	< 0.001	-0.46
							II vs. III	749.500	0.467	-0.10
Emotional	1.60	1.20	2.80	1.20	3.30	2.04	I vs. II	972.000	< 0.001	-0.52
Inhibition							I vs. III	1414.000	< 0.001	-0.61
							II vs. III	854.500	0.113	-0.21
Unrelenting	2.80	1.30	3.60	1.55	3.40	2.75	I vs. II	926.000	0.003	-0.41
Standards							I vs. III	1057.000	0.172	-0.17
							II vs. III	599.500	0.271	0.15
Entitlement	1.40	0.80	1.60	0.90	1.80	1.35	I vs. II	776.000	0.178	-0.18
							I vs. III	1142.500	0.033	-0.27
							II vs. III	796.000	0.330	-0.13
Insufficient	1.80	1.00	2.40	1.35	3.00	1.20	I vs. II	871.000	0.017	-0.33
Self-Control							I vs. III	1452.000	< 0.001	-0.61
							II vs. III	913.500	0.027	-0.30
Approval-	2.80	2.00	3.40	1.60	3.20	1.94	I vs. II	804.000	0.099	-0.22
Seeking							I vs. III	1090.000	0.098	-0.21
							II vs. III	701.000	0.975	0.00
Negativity/	2.00	1.70	4.20	1.90	4.20	1.35	I vs. II	1053.500	< 0.001	-0.61
Pessimism							I vs. III	1576.000	< 0.001	-0.75
							II vs. III	811.000	0.259	-0.15
Punitiveness	2.00	1.40	3.50	1.40	3.60	2.55	I vs. II	1028.500	< 0.001	-0.61
							I vs. III	1369.500	< 0.001	-0.56
							II vs. III	727.500	0.804	-0.03

^a^Mann-Whitney U-test; ^b^Cliff’s delta; ≥0.15 small, ≥0.33 medium, ≥0.48 large

Considering the median values of the EMSs on a continuum from no self-harm to only self-harm thoughts, and onwards to both self-harm thoughts and behavior, the higher the scores for the EMSs, the more severe the self-harm manifestations were. The only exceptions to this were Unrelenting Standards (the SHT group had slightly higher median values than the SHTB group), Approval-Seeking (the SHT group having slightly higher median values than the SHTB group) and Negativity/Pessimism (the medians of the SHT and SHTB groups were equal).

### Associations of EMSs with adolescents’ non-suicidal self-harm functions

Females had significantly higher Internal Emotional Regulation function scores than males (Median = 2.06, IQR = 1.09 vs. Median = 1.00, IQR = 0.75, p = 0.001), but regarding other functions, there were no significant gender differences. Between patient groups, living conditions or the use of tobacco products were not significantly associated with the functions. Regarding the occupations of participants’ parents, those with both parents working (Median = 1.17, IQR = 1.33) had higher External Emotional Regulation scores than those with one parent/neither working (Median = 1.00, IQR = 1.00, p = 0.049). Regarding participants’ occupation, the Internal Emotional Regulation scores differed significantly for the groups: on (Lower secondary school pupils Median = 1.75, IQR = 1.63; Upper secondary or higher education students Median = 2.25, IQR = 1.06; Other: Median = 1.50, IQR = 1.44, p = 0.036. Additionally, several EMSs had significant correlations with Internal Emotional Regulation, Social influence, External Emotional Regulation and Sensation Seeking functions of adolescents’ non-suicidal self-injury (Table 4). Age had no significant correlations with any of the functions, but the OASIS total score correlated significantly with Internal Emotional Regulation.

**Table 4:** Spearman’s correlation coefficients of YSQ scales, OSI-F functions, participants’ age and OASIS total score

	ED	AB	MA	SI	DS	FA	DI	VH	EM	SB	SS	EI	US	ET	IS	AS	NP	PU	Age	OAS	IER	SIE	EER	SSE
ED	-	0.618**	0.696**	0.714**	0.697**	0.629**	0.559**	0.534**	0.427**	0.673**	0.241**	0.564**	0.233**	0.294**	0.490**	0.271**	0.593**	0.545**	0.344**	0.578**	0.243	0.229	0.029	− 0.080
AB		-	0.613**	0.593**	0.667**	0.632**	0.482**	0.563**	0.326**	0.627**	0.434**	0.350**	0.272**	0.259**	0.471**	0.425**	0.670**	0.537**	0.355**	0.643**	0.378**	− 0.012	0.284*	0.248
MA			-	0.697**	0.681**	0.613**	0.552**	0.644**	0.453**	0.670**	0.377**	0.566**	0.287**	0.308**	0.493**	0.369**	0.660**	0.602**	0.321**	0.602**	0.268	0.185	0.101	0.107
SI				-	0.754**	0.679**	0.587**	0.602**	0.430**	0.690**	0.244**	0.590**	0.173**	0.334**	0.589**	0.341**	0.624**	0.558**	0.272**	0.597**	0.095	0.094	0.133	0.150
DS					-	0.743**	0.592**	0.566**	0.379**	0.746**	0.301**	0.558**	0.266**	0.266**	0.515**	0.356**	0.662**	0.644**	0.266**	0.601**	0.309*	− 0.007	0.136	0.110
FA						-	0.673**	0.561**	0.399**	0.644**	0.271**	0.497**	0.187**	0.226**	0.603**	0.327**	0.689**	0.562**	0.344**	0.598**	0.324*	− 0.027	0.086	0.126
DI							-	0.583**	0.491**	0.621**	0.113*	0.423**	0.146*	0.368**	0.623**	0.255**	0.557**	0.450**	0.161**	0.537**	0.214	0.137	0.185	0.015
VH								-	0.428**	0.623**	0.298**	0.507**	0.299**	0.354**	0.549**	0.351**	0.717**	0.532**	0.195**	0.603**	0.206	0.032	0.064	− 0.043
EM									-	0.454**	0.137*	0.397**	0.177**	0.375**	0.442**	0.228**	0.411**	0.355**	0.079	0.335**	− 0.080	0.024	0.042	0.035
SB										-	0.313**	0.515**	0.257**	0.280**	0.563**	0.412**	0.641**	0.598**	0.222**	0.633**	0.311*	− 0.185	0.083	0.084
SS											-	0.202**	0.387**	0.063	0.213**	0.445**	0.422**	0.420**	0.236**	0.330**	0.374**	0.123	0.136	0.133
EI												-	0.236**	0.377**	0.494**	0.250**	0.514**	0.442**	0.217**	0.451**	− 0.089	0.139	− 0.051	0.068
US													-	0.128*	0.215**	0.447**	0.374**	0.530**	0.239**	0.284**	0.298*	0.277*	0.380**	0.313*
ET														-	0.523**	0.235**	0.315**	0.276**	0.054	0.190**	− 0.177	0.370**	− 0.021	0.239
IS															-	0.392**	0.611**	0.525**	0.275**	0.482**	0.139	− 0.003	0.068	0.036
AS																-	0.467**	0.506**	0.256**	0.298**	0.119	0.296*	0.031	0.272*
NP																	-	0.690**	0.262**	0.664**	0.227	− 0.009	0.163	− 0.059
PU																		-	0.219**	0.538**	0.223	0.099	0.218	0.060
Age																			-	0.354**	0.136	0.168	− 0.055	0.046
OAS																				-	0.422**	0.036	0.221	− 0.041
IER																					-	0.142	0.432**	0.232
SIE																						-	0.249	0.253
EER																							-	0.310*
SSE																								-

YSQ scales: ED = Emotional Deprivation, AB = Abandonment, MA = Mistrust/Abuse, SI = Social Isolation, DS = Defectiveness, FA = Failure, DI = Dependence, VH = Vulnerability, EM = Enmeshment, SB = Subjugation, SS = Self-Sacrifice, EI = Emotional Inhibition, US = Unrelenting Standards, ET = Entitlement, IS = Insufficient Self-Control, AS = Approval-Seeking, NP = Negativity/Pessimism, PU = Punitiveness

OSI-F-factors: IER = Internal Emotional Regulation, SIE = Social Influence, EER = External Emotional Regulation, SSE = Sensation Seeking

Age: Participants’ age

OAS = Participants’ OASIS total score

*p < 0.05

**p < 0.01

When the confounders were considered, Self-Sacrifice was the only EMS that had a significant explanatory effect for Internal Emotional Regulation (p = 0.021) (Table 5). Regarding Social Influence function, after controlling for the confounders, none of the EMSs were significant explanatory variables for the variations in the function (Table 6). For External Emotional Regulation, Abandonment was a significant independent variable explaining the variation in the function (p = 0.040), as well as Unrelenting Standards (p = 0.012). Finally, either Unrelenting Standards or Approval-Seeking were not significantly associated with the Sensation Seeking function.

### Significance of EMSs to addictive features of adolescents’ non-suicidal self-injury

Comparing the Not Addicted and Addicted to self-harm groups, significant differences were found for Abandonment (Median = 3.20, IQR = 2.00 vs. Median = 4.10, IQR = 1.60, p = 0.028), Dependence (Median = 2.20, IQR = 0.80 vs. Median = 3.00, IQR = 1.85, p = 0.033) and Punitiveness (Median = 3.20, IQR = 2.20 vs. Median = 4.10, IQR = 1.95, p = 0.037) EMSs, the scores being higher for the Addicted group. Regarding the background variables, only occupation differed significantly (χ^2^ [2] = 6.759, p = 0.034) in the Addictive function categories. In the logistic regression analyses, Abandonment and Dependence EMSs were significantly associated with addictive features (Table 7).

**Table 5:** Multivariate associations of the Early Maladaptive Schemas (EMSs) and confounders with Internal Emotional Regulation

	F	df	p^a^		F	df	p^a^		F	df	p^a^		F	df	p^a^		F	df	p^a^		F	df	p^a^
Abandonment	3.335	1	0.075	Defectiveness	0.800	1	0.377	Failure	3.630	1	0.064	Subjugation	1.113	1	0.298	Self-Sacrifice	5.759	1	0.021	Unrelenting Standards	0.940	1	0.338
OASIS total score	4.724	1	0.036	OASIS total score	4.383	1	0.043	OASIS total score	3.308	1	0.076	OASIS total score	3.394	1	0.073	OASIS total score	4.641	1	0.037	OASIS total score	3.763	1	0.059
Gender	1.912	1	0.174	Gender	2.899	1	0.096	Gender	2.701	1	0.108	Gender	3.586	1	0.065	Gender	4.798	1	0.034	Gender	3.326	1	0.075
Occupation	2.142	2	0.130	Occupation	1.718	2	0.192	Occupation	2.325	2	0.111	Occupation	1.491	2	0.237	Occupation	1.213	2	0.308	Occupation	1.644	2	0.206
Gender * Occupation	1.223	2	0.305	Gender * Occupation	0.750	2	0.479	Gender * Occupation	1.096	2	0.344	Gender * Occupation	0.528	2	0.594	Gender * Occupation	0.580	2	0.565	Gender * Occupation	0.682	2	0.511
Error		41		Error		40		Error		41		Error		41		Error		41		Error		41	
Adjusted R Squared	0.341			Adjusted R Squared	0.299			Adjusted R Squared	0.345			Adjusted R Squared	0.306			Adjusted R Squared	0.375			Adjusted R Squared	0.303		

^a^General Linear Model: Univariate Analysis of Variance

F = Fisher’s F-test

OASIS = Overall Anxiety Severity and Impairment Scale

**Table 6:** Multivariate associations of the Early Maladaptive Schemas and confounders with the other self-injury functions

Social Influence^b^																
	F		df	p^a^			F		df	p^a^			F	df		p^a^
Unrelenting Standards	2.933		1	0.095	Entitlement		2.646		1	0.112	Approval-Seeking		1.941	1		0.172
Error			38		Error				38		Error			38		
Adjusted R Squared	0.047				Adjusted R Squared		0.040				Adjusted R Squared		0.024			
External Emotional Regulation^b^																
		F		df		p^a^						F	df		p^a^	
Abandonment		4.467		1		0.040		Unrelenting Standards				6.937	1		0.012	
Occupation of parents		2.378		1		0.130		Occupation of parents				3.827	1		0.057	
Error				45				Error					45			
Adjusted R Squared		0.102						Adjusted R Squared				0.145				
Sensation Seeking^b^																
		F		df		p^a^						F	df		p^a^	
Unrelenting Standards		1.836		1		0.186		Approval-Seeking				1.765	1		0.195	
Error				28				Error					28			
Adjusted R Squared		0.028						Adjusted R Squared				0.026				

^a^General Linear Model: Univariate Analysis of Variance

^b^Log-transformed variable

F = Fisher’s F-test

**Table 7:** Logistic regression analyses for Addiction Component using correlative Early Maladaptive Schemas and confounders

	B	Wald	p^a^	Exp (B)		B	Wald	p^a^	Exp (B)		B	Wald	p^a^	Exp (B)
Abandonment	0.543	4.102	0.043	1.721	Dependence	0.800	5.027	0.025	2.225	Punitiveness	0.423	3.075	0.079	1.527
Other occupational situation		5.742	0.057		Other occupational situation		6.924	0.031		Other occupational situation		5.434	0.066	
Lower secondary school pupil	1.972	5.447	0.020	7.184	Lower secondary school pupil	2.185	5.832	0.016	8.888	Lower secondary school pupil	1.853	4.971	0.026	6.376
Upper secondary school/Higher education student	1.585	3.538	0.060	4.879	Upper secondary school /Higher education student	2.148	5.660	0.017	8.567	Upper secondary school /Higher education student	1.616	3.771	0.052	5.035
R^2^	0.272					0.321					0.245			
Model fitting data^b^	χ^2^ = 11.611 df = 3 p = 0.009					χ^2^ = 13.756 df = 3 p = 0.003					χ^2^ = 10.350 df = 3 p = 0.016			

^a^Logistic Regression Analysis

^b^Omnibus Tests of Model Coefficients

Exp (B) = Odds Ratio for participants to belong in Addiction - category (Addiction – No Addiction) regarding Addiction Component

R^2^ = Nagelkerke Pseudo-R Square

## Discussion

The main results in the present study were that EMSs were significantly associated with participants’ self-harm and its severity. When comparing participants with no self-harm to those with only self-harm thoughts, and to those with both self-harm thoughts and behavior, the participants without self-harm had the lowest EMS scores. The participants with the most severe manifestations of self-harm had the highest scores on almost all EMSs. Regarding the self-injury functions, Internal Emotional Regulation was associated with Self-Sacrifice EMS, and External Emotional Regulation with Abandonment and Unrelenting Standards EMSs. In addition, being addicted to self-injury was associated with Abandonment and Dependence EMSs.

### The associations of EMSs with self-harm thoughts and behavior in adolescents

Almost all EMSs were significantly different for participants without self-harm thoughts or behavior compared to those who had self-harm (SH) thoughts only. Additionally, also the differences between the participants without SH thoughts and those with both self-harm thoughts and behavior were significant. Moreover, comparing the groups with SH thoughts and those with both SH thoughts and behaviors, seven EMSs were significantly higher for the latter. Overall, it appeared that the strength of the EMSs was a more significant factor than the specific EMSs in differentiating the severity of SH.

Considering previous research, it is known that self-harm and self-injury are associated with intrapersonal and interpersonal factors [15, 21], emotion regulation difficulties [22, 24], and diverse emotionally painful thoughts and experiences [13, 14]. It is noteworthy that at the core of EMSs are various adverse experiences regarding, and beliefs formulated about, emotions and relationships, as well as oneself [31]. Thus, reflecting on the present results, if the EMSs are very widespread and intense in the mind of an individual, the individuals’ emotional pain might be more intense overall when they face difficult experiences in life. In addition, the maladaptive means to cope with the emotions might strengthen them as well. Therefore, this could make individuals more vulnerable to self-harm.

### The associations of EMSs with functions of adolescents’ non-suicidal self-injury behavior

Considering the results for non-suicidal self-injury (NSSI) functions from the multivariate analyses, Self-Sacrifice was independently associated with the Internal Emotional Regulation function. In the OSI-F questionnaire, Internal Emotional Regulation consists of coping with experiences regarding, for example, loneliness, sadness, self-punishment, distraction from unpleasant experiences and discontinuing suicidal thoughts and actions [27, 28, 43]. Many of those experiences could be seen reflecting different forms of being estranged from, or distancing oneself from, emotions in emotionally-charged experiences. In addition, as it has been shown in previous research, the association of emotion regulation difficulties and self-injury exists [22, 26]. Thus, considering the present results, those individuals with EMSs entailing a tendency to sacrifice their own emotional needs for the emotional needs of others [31], may try to distance themselves from their emotions. They may also try to cope with emotional estrangement in unhealthy ways, such as self-injuring.

None of the EMSs were independently associated with the Social Influence or Sensation Seeking functions, but Abandonment and Unrelenting Standards independently explained variation in the External Emotional Regulation function. Trying to cope with feelings of anger, frustration and tension are at the core of the External Emotional Regulation function in OSI-F [27, 28, 43]. The previous studies have shown the associations between self-injury and the emotion regulation aspects [22, 26, 30]. Hence, reflecting the present results, those individuals with the EMSs entailing the experience of abandonment and unreasonable demands towards themselves [31] may have difficulties in coping with the aforementioned emotions in particular. As such, this could create vulnerability to self-injuring in these individuals.

### The associations of EMSs with addictive features of adolescents’ non-suicidal self-injury behavior

Abandonment and Dependence were independently associated with individuals addicted to NSSI. This finding was interesting in the sense that the individuals addicted to NSSI seem to have a tendency to experience both being abandoned and an elevated need for others’ emotional support in coping, when considering the core ideas of those EMSs [31]. Previous studies have shown that both emotion regulation issues [22, 25] and interpersonal and intrapersonal factors are associated with self-injury [15, 21]. Thus, considering the present results, the mental conflict described can create rather intense emotional pain that the individuals try to alleviate with NSSI. This may also reflect a specific vulnerability in these individuals, as the individuals who are addicted to NSSI might be especially lacking in both available emotional support and the skills to regulate their emotions adaptively.

The results indicating associations between the EMSs with adolescents’ self-harm are in line with previous studies [33, 34]. However, in the present study, the strength of the EMSs seemed to be especially important in these associations. The results are also in line with previous studies that have not investigated EMSs, but which have studied other ideational characteristics that individuals may have in association with urges to self-harm. For example, ideation regarding self-criticism and self-judgement [13, 14] were also apparent in association with NSSI in the present results when reflecting the functions of NSSI. Furthermore, as it has been indicated in previous research [15, 21, 35], the participants seemed to engage in NSSI for various functions.

The results of the present study are promising, but considering the limitations of the study, they should be considered with caution. The rather small number of participants particularly in the group comparisons may have influenced the analyses and results. Although the sample was, for the most part, representative, the participation rate was higher among females and those who had no experience of using tobacco products. Although the study sample comprised adolescents, the participants’ age range was large and thus, also their developmental stages were varied. Additionally, regarding the first study question, due to the large number of significant differences between the three groups, multivariate models were not built for the separate EMSs, which should be taken into account when considering the results. The analyses were also not corrected for multiple testing. Regarding the second and third study questions, the significance of EMSs on the functions and addictive features of NSSI might have gained more support if the number of participants would have been greater. Another limitation of this study was that a translated but not psychometrically studied or validated version of OSI-F was used. Furthermore, in the present study, the Cronbach’s alpha values for OSI-F function factors were rather small. This may have affected the results, and thus conclusions made from the results regarding the functions should be considered with caution. In future, it would be important to confirm the psychometric properties of the OSI-F translation. In addition, the fact that the YSQ and OASIS questionnaires were completed at a different time point than the OSI-F questionnaire may have affected the results. It is plausible that the OASIS scores fluctuated between the two time-points, but it should be noted that, at least in adults, EMSs have been shown to be quite stable [46]. Additionally, several questions in the OSI-F questionnaire assess self-harm/self-injury that has happened in the past and thus, possibly bridges this gap to some extent.

More research to confirm the present findings is needed, but in future, assessing the EMSs and their strength may help to identify those individuals who have a greater risk for self-harm in clinical practice. In addition, better understanding of the different reasons for self-injury among adolescents may help in developing more efficient treatment methods for the behavior. For example, individuals who tend to sacrifice their own emotional needs for others and who feel estranged from their painful emotions, may benefit from treatment approaches that address their underlying fears of facing their emotions more directly and support that they keep healthy emotional boundaries with others. In comparison, those individuals who feel abandoned, are demanding or experience feelings of anger, might benefit from approaches that help them to cope with their anger in constructive ways.

In addition, it is also be possible that some self-injuring adolescents may benefit from treatments related to addictions. These individuals might also benefit from approaches that address both on an emotional and social level their heightened need for support from others and their experiences of being abandoned. Additionally, future studies could explore whether individuals’ susceptibility to dependence on something else, e.g., substances, increase their risk to be addicted to self-injury. Future studies should also address other possible psychological concepts and measures in conjunction with EMSs, and also include a more thorough assessment of psychological stressors and coping strategies. Additionally, studies in larger samples should be conducted to verify these results and the investigation of these factors in conjunction with developmental aspects of adolescence could be particularly fruitful.

## Conclusions

The present study findings give support to the existence of meaningful associations of EMSs with self-harm and self-injury in adolescents. As most EMSs associated with self-harm, and the strength of the EMSs had a connection with the severity of adolescents’ self-harm, it could be that as the maladaptive emotional-cognitive functioning intensifies, the risk for maladaptive coping mechanisms increases. The associations of certain EMSs with Internal Emotional Regulation and External Emotional Regulation functions highlight a possibility that different functions of self-injury may indeed be associated with, or possibly even triggered by, different maladaptive emotional-cognitive patterns. In addition, given the associations of certain EMSs with addiction to self-injury behavior, certain maladaptive emotional-cognitive patterns may associate with an increased risk of adolescents becoming addicted to behavior that is fundamentally detrimental for them.

As the EMSs as well as self-harm and self-injury all reflect psychological and behavioral pathologies, the present findings that the majority of EMSs as well as their strength were associated with self-harm seem understandable. In this regard, the present findings highlight the importance of paying attention to maladaptive structures and functioning of psyche, not only the apparent psychological symptoms or behavior, when considering the risks for and treatment of self-harm. As the associations between EMSs and self-harm were so extensive, a question arises whether adolescents are especially vulnerable to developing self-harm when the structure and functioning of their psyche develop maladaptive features.

Regarding self-injury functions and addiction, the EMSs of Self-Sacrifice, Abandonment, Unrelenting Standards and Dependence emerged as significant in the present findings. These findings are interesting especially when considered from the viewpoint of adolescence. Sacrificing and invalidating one’s own emotional needs and having unreasonably high demands of oneself, as well as feeling abandoned by or excessively dependent on others, are all problematic premises for the adolescent psychological development. Based on these premises, the development of a balanced identity, psychological requirements of separating from parents, and adaptation to the psychological demands of adulthood might be difficult. In this regard, a question arises from the present results whether these EMSs together with self-harm and self-injury could represent, on one hand, an extreme crisis in adolescent psychological development.

## Data Availability

The data that support the findings of this study are not openly available due to reasons of sensitivity. However, they are available from the corresponding author (Max Karukivi) upon reasonable request.

## Abbreviations

EMS(s): Early Maladaptive Schema(s)
NSSI: Non-suicidal self-injury
SH: Self-harm
OASIS: Overall anxiety severity and impairment scale
YSQ: Young schema questionnaire – short form 2 – Extended
OSI-F: Ottawa self-injury inventory – functions (v3.1)
NSH: No self-harm
SHT: Self-harm thoughts but no behavior
SHTB: Self-harm thoughts and behavior
IQR: Interquartiles
GLM: General linear model
ED: Emotional Deprivation
AB: Abandonment
MA: Mistrust/Abuse
SI: Social Isolation
DS: Defectiveness
FA: Failure
DI: Dependence
VH: Vulnerability
EM: Enmeshment
SB: Subjugation
SS: Self-Sacrifice
EI: Emotional Inhibition
US: Unrelenting Standards
ET: Entitlement
IS: Insufficient Self-Control
AS: Approval-Seeking
NP: Negativity/Pessimism
PU: Punitiveness
IER: Internal Emotional Regulation
SIE: Social Influence
EER: External Emotional Regulation
SSE: Sensation Seeking
